# Reducing Early Antibiotic Use: A Quality Improvement Initiative in a Level III Neonatal Intensive Care Unit

**DOI:** 10.3389/fped.2022.913175

**Published:** 2022-05-31

**Authors:** Catalina Morales-Betancourt, Javier De la Cruz-Bértolo, Bárbara Muñoz-Amat, Elena Bergón-Sendín, Carmen Pallás-Alonso

**Affiliations:** ^1^Department of Neonatology, 12 de Octubre University Hospital, Madrid, Spain; ^2^Health Research Institute Imas 12, 12 de Octubre University Hospital, Madrid, Spain; ^3^Primary Care Interventions to Prevent Maternal and Child Chronic Diseases of Perinatal and Developmental Origin (RICORS), Instituto de Salud Carlos III, Madrid, Spain

**Keywords:** quality improvement, antibiotic stewardship, neonatal intensive care, very low birth weight, process indicators

## Abstract

Antibiotic burden is a critical issue in neonatal intensive care units (NICU) and antibiotic use is considered a quality indicator of neonatal care. Our aim was to optimize antibiotic use through a quality improvement (QI) initiative that included revision of departmental protocols and implementation of a surveillance system based on process indicators.

**Methods:**

This is descriptive study of a cohort of all very low birth weight (VLBW) infants admitted to the NICU from 2014 to 2019. A series of QI interventions were made during the study period and included departmental protocols and the implementation of a surveillance system based on process indicators. The primary outcome was the percentage of VLBW infants who had received **early antibiotics** (ampicillin, gentamicin, or cefotaxime on the day of birth or day 1 or 2 after birth), **antibiotics for longer than 3 days** (despite negative blood culture), or **no antibiotics**.

**Results:**

During the study period, a significant relative reduction was seen in the proportion of VLBW infants administered early antibiotics (46%; *p* < 0.01) and in infants provided antibiotics for longer than 3 days (90%; *p* < 0.01). Additionally, the percentage of VLBW with “no antibiotics” during their NICU stay increased fivefold (6 to 30%; *p* < 0.001).

**Conclusions:**

In our NICU, the implementation of a QI initiative that is based on affordable methods to track process indicators and evaluate the results led into a significant reduction in antibiotic exposure in VLBW infants. This approach is easy to implement in other NICUs as well.

## Introduction

Antibiotics are the most frequent drugs prescribed in the NICUs (1), and while there are situations where they are indeed lifesaving, it has been estimated that 20–50% of antibiotic prescriptions in the NICUs are inappropriate (2). Antibiotic use in the early stages of life is associated with perturbation of the microbiome (3), emergence of multidrug-resistant organisms (4), and adverse neonatal outcomes in term and preterm infants. Antibiotic exposure during the first days of life in VLBW infants is also associated with increased risk of necrotizing enterocolitis (NEC), late onset sepsis (LOS), bronchopulmonary dysplasia (BPD), and death (5–10). Hence, European guidelines on the prudent use of antimicrobials in human health have proposed principles that not only promote their judicious use but also present measures to optimize antimicrobial prescribing and stewardship, including “use of local guidelines, monitor and audit appropriate use of antimicrobials through quantity and quality indicators and systems for monitoring these indicators, and ensure regular feedback of the results to prescribers (11).” Similarly, the Centers for Disease Control and Prevention (United States of America) and the American Academy of Pediatrics recommend the use of strategies that optimize antibiotic use (12, 13).

Notably, NICUs that included some of these measures described in antibiotic stewardship programs and quality improvement (QI) projects have obtained significant reductions in antibiotic use (13–17). For example, the Pediatrix Group, through the 100,000 Babies Campaign, identified key driver processes to improve multiple domains of care in a large neonatal network within a QI program. One of the key drivers identified was related to medication, wherein the use of ampicillin on the day of birth or days 1 or 2 after birth, and use of ampicillin for longer than 3 days despite negative cultures were designated as goal indicators. The implementation of measures to optimize antibiotic use resulted in participant NICUs improving their results in terms of doses of antibiotic (18).

Although monitoring and measuring antibiotic use are quality indicators in the neonatal setting, there is great variability in their measurement. Moreover, there is uncertainty about the optimal rate of antibiotic use in NICUs (19, 20). Hence, NICUs typically update and modify their clinical practices based on new data and available evidence. However, tracking these changes is challenging as they are not typically implemented as part of a research study.

Aiming to follow international recommendations and best practices for antibiotic use, our NICU implemented a few changes, both in clinical practice and related process indicators. Here, we describe antibiotic use as a process indicator and our primary objective was to assess changes in the following variables between 2014–2019: (1) frequency of antibiotic use in the first three days of life in very low birth weight (VLBW) infants; (2) its continuation beyond the third day of life despite negative blood culture; and (3) proportion of VLBW infants who were not administered antibiotics from birth till hospital discharge. Our secondary objective was to evaluate variations in critical neonatal outcomes over the study period as an indicator of the safety of the changes.

## Methods

### Setting

The study was performed at the NICU of the 12 de Octubre University Hospital, Madrid, Spain. The NICU is a level III C unit, i.e., maximum complexity level as per the Spanish Society of Neonatology. It is also a regional and national referral center with 44 beds, including 19 intensive care beds. The unit cares for surgical and non-surgical preterm neonates and term neonates who are sick, with nearly 800 admissions annually, about 100 of whom are VLBW.

Clinical practice in the unit is based on departmental guidelines, with documents being evidence-based and updated periodically. Guidelines are approved as per internal standard operating procedures. Each guideline and its updates are personally notified to the staff by e-mail and are presented during academic meetings with neonatologists, pediatric residents, research nurses, and NICU nurses. All guidelines are available on paper and in electronic form on the NICU's cloud platform. During the study period, all medical records and drug prescriptions were registered in paper charts.

If antibiotics are indicated by neonatologist, one sample for blood culture (at least 1 cc) is obtained before the administration. Blood culture is collected by a nurse, from venipuncture and or during insertion of peripherally inserted central catheter. It is only obtained by the neonatologist if its during umbilical catheter insertion. The volume obtained is registered in medical charts to assess its reliability. Blood cultures are immediately delivered at the microbiology department, where optimized enriched culture media with antimicrobial properties and continuous-read detection systems are used. In the scenario of EOS, our unit use C-reactive protein as a biomarker, is obtained at discretion of the attending neonatologist, the decision of starting antibiotics it is not made based on C-reactive protein values.

### Clinical Practice Changes

The NICU has made two updates to the VLBW antibiotic use policy:

#### Early Suspension

From December 2014, the duration of early antibiotic use in VLBW infants was shortened based on emerging literature. This clinical practice was notified as an update to the guidelines on the care of VLBW in March 2015, which stated that antibiotics (ampicillin, gentamicin, or cefotaxime), started at birth, should be suspended at 72 h if blood cultures were negative. Additionally, the time of stopping must be included in the discharge report.

#### No Antibiotics for VLBW Infants at Low Risk of Early Onset Sepsis (EOS)

A guideline regarding antibiotic therapy at birth in VLBW infants was developed between November 2017 and January of 2018, and was presented to the NICU by the end of January 2018. The document emphasized that antibiotics should not be started after birth if all of the following criteria were met, namely, birth by cesarean delivery, absence of signs of chorioamnionitis, absence of any rupture of membranes before delivery.

### Assessment of Clinical Practice Changes

Since 2017, our NICU adopted certain process indicators used by the Pediatrix Group (18) to evaluate performance in different areas, and an annual self-assessment report is prepared and communicated to the staff. Through process indicators related to antibiotic use, we have established a surveillance system to monitor the use of antibiotics in VLBW infants. This surveillance system was initiated on January 2017 and the first feedback to the unit was provided on March 2018. We also monitored mortality and morbidity outcomes to detect any adverse events related to these changes in clinical practice.

#### Process Indicators

**Early antibiotics, antibiotics for longer than 3 days**, and **no antibiotics** were used as indicators (definitions below).

#### Surveillance System

Since 2017, surveillance of the indicators included the following elements:

- Daily audit: During clinical rounds, the antibiotic status of each infant in the NICU is discussed with consultant neonatologists, pediatric residents, and the Heads of the Department.
- Patient report: At discharge, the chief of the department verifies the medical records and the antibiotics administered to the infant from birth until discharge. The discharge report includes information on whether the infant had received **early antibiotics** (yes/no), **antibiotics for longer than 3 day**s (yes/no and reason), or **no antibiotics** (yes/no).
- Coding: At discharge, all indicators were checked, coded, and included in the database of the unit by the chief of the department.
- Annual feedback: Annually, the chief of the department analyzed the NICU's database and compared process indicators with data from the Pediatrix group. The results were evaluated, communicated to the staff, and sent to the hospital's director, as part of the self-assessment report.

### Study Design

This was a follow-up study of a cohort of all inborn VLBW infants, with birth weight (BW) between 500–1500 g, admitted since birth to the NICU, 12 de Octubre University Hospital, from 01 January 2014, to 31 December 2019. Neonates with malformations incompatible with life were excluded. The study period included a retrospective data collection period from 2014 to 2016, before the start of the surveillance system. For this subcohort, data on process indicators were retrieved from unit medical records. A prospective data collection system was used for the period 2017–2019, i.e., since the implementation of the surveillance system in 2017.

### Measures

#### Primary Outcomes

We used the following process indicators to estimate the percentage of VLBW infants who had received **early antibiotics, antibiotics for longer than 3 days**, or **no antibiotics**. The definitions are identical to those used by the Pediatrix Group, as this ensures consistency with existing data and enables comparison with other units. **Early antibiotics** was defined as the proportion of infants exposed to ampicillin, gentamicin, or cefotaxime on the day of birth or on days 1 or 2 after birth. **Antibiotics for longer than 3 days** indicated the proportion of infants who were administered ampicillin, gentamicin, or cefotaxime for longer than 3 days despite negative blood culture (18). **No antibiotics** was defined as the percentage of infants discharged without any prescribed antibiotics during their stay in the NICU.

#### Secondary Outcomes

We defined early mortality as death before 72 h of life and late mortality as death between 72 h of life and discharge. BPD was defined as requiring oxygen at 36 weeks' postmenstrual age or at discharge, whichever came first; retinopathy of prematurity (ROP) stage ≥ III or those that needed treatment; NEC Bell's Stage ≥ II; brain injury as intraventricular hemorrhage (grade three or associated with hemorrhagic infarction). To assess morbidity and mortality, we used a composite outcome of late mortality and one of four major morbidities (BPD, ROP, NEC, and brain injury).

EOS was sepsis diagnosed by blood culture within 3 days of birth while LOS was defined as sepsis occurring after the third day of life with positive blood culture. Severity of illness was assessed using the clinical risk index for babies (CRIB) (21), which is a validated predictor of mortality in infants born at ≤ 32 weeks' gestation that incorporates information on gestational age (GA), BW, sex, initial base deficit, and oxygen requirements.

An increase in the length of NICU stay, due to an adverse event attributable to changes in clinical practice was evaluated as a safety measure.

### Data Collection

Data was collected from clinical records and the unit database. Variables such as prenatal history, GA, BW, APGAR score, delivery room resuscitation, CRIB, and the evaluated measures were exported to an anonymous database.

### Statistical Analyses

This manuscript adheres to the standards for QI excellence (SQUIRE) 2.0 guidelines (22, 23). Descriptive statistics included mean values and standard deviation (SD) for normally distributed continuous variables, and absolute and relative frequencies for categorical variables. Trends of antibiotic use and of characteristics for the study period (2014–2019) were analyzed using the Cochran-Armitage Trend test. Change over time in continuous variables was assessed using analysis of variance (ANOVA). A *p*-value of < 0.05 was considered statistically significant. SAS/STAT software, Version 9.4 (SAS Institute Inc., Cary, NC) was used for analyses.

Statistical process control charts (SPC) were created (QI Macros, KnowWare International, Inc, Denver, CO) to display and analyze data over time. The following rules were used to determine special cause variation (SCV): one point outside the upper or lower control limits, two of three points beyond 2 SD from the mean on the same side of the center line (CL), four of five successive points beyond 1 SD from the mean on the same side of the CL, eight successive points on the same side of the CL, or six successive increasing or decreasing points (24).

## Results

We identified 638 infants with BW between 500 and 1,500 g during the study period. Of these, 50 were excluded as 42 were born in other maternities and 8 had insufficient data in their medical charts. Thus, data from 588 VLBW infants were included in the analysis. This cohort had a mean GA of 28.6 weeks (SD 2.8), and a mean BW of 1,042 g (SD 282).

### Patient Characteristics

Infant and maternal characteristics are shown in Table 1. No differences over time were observed in GA, BW, or early mortality (trend test, all *p*-values > 0.05).

**Table 1:** Infant and antibiotic characteristics of VLBW (< 1500 g), 2014 to 2019.

	**2014**	**2015**	**2016**	**2017**	**2018**	**2019**	**2014–2019**	***p*-value^**a**^**
	***N*** **=** **101** ***N*** **(%)**	***N*** **=** **94** ***N*** **(%)**	***N*** **=** **95** ***N*** **(%)**	***N*** **=** **98** ***N*** **(%)**	***N*** **=** **104** ***n*** **(%)**	***N*** **=** **96** ***n*** **(%)**	***N*** **=** **588** ***n*** **(%)**	
*Infant characteristics*								
Gestational age (wk), mean (SD)	29 (2.7)	29 (2.7)	28 (2.8)	28 (2.6)	28 (2.5)	28 (3.2)	28.6 (2.8)	0.5
Birth weight (g), mean (SD)	1036 (294)	1078 (285)	1020 (261)	1029 (281)	1086 (273)	1000 (274)	1042 (282)	0.3
Male	48 (48%)	47 (50%)	49 (51%)	52 (53%)	67 (64%)	51 (53%)	314 (53%)	0.01
Multiple gestation	27 (27%)	41 (44%)	29 (31%)	36 (37%)	43 (41%)	28 (29%)	204 (35%)	0.12
Antenatal steroids	89 (88%)	79 (85%)	86 (91%)	96 (98%)	97 (93%)	93 (97%)	540 (92%)	0.01
Eutocic delivery	23 (23%)	15 (16%)	24 (25%)	25 (26%)	24 (23%)	27 (28%)	138 (23%)	0.4
Low birth weight	37 (37%)	29 (31%)	27 (28%)	34 (35%)	31 (30%)	38 (40%)	196 (33%)	0.5
CRIB (SD)	4 (4)	3 (4)	4 (4)	4 (4)	4 (4)	4 (4)	3.7 (4)	0.4
Early mortality^b^	2 (2%)	1 (1%)	2 (2%)	0 (0%)	3 (3%)	4 (4%)	12 (2%)	0.2
EOS	2 (2%)	4 (4%)	2 (2%)	1 (1%)	2 (2%)	1(1%)	12 (2%)	0.5
*Antibiotic characteristics*								
No antibiotics^c^	6 (6%)	14 (15%)	19 (20%)	25 (26%)	43 (41%)	29 (30%)	136 (23%)	<0.001
Early antibiotics^d^	92 (91%)	73 (78%)	72 (76%)	55 (56%)	56 (54%)	47 (49%)	395 (67%)	<0.001
Antibiotics during >3 days (*n* = 571)^e^	52/98 (53%)	21/90 (23%)	20/91 (22%)	24/97 (24%)	13/100 (13%)	5/91 (5%)	135 (24%)	<0.001

*WK, weeks; g, grams; SD, standard deviation; EOS, early onset sepsis. ^a^The p-value corresponds to a trend analysis for categorical variables and an analysis of variance for continuous variables for the period 2014-2019. ^b^Early Mortality: death before 72 h. ^c^No antibiotics: percentage of infants discharged without receiving antibiotics during the stay in the unit. ^d^ Early antibiotics: proportion of infants exposed to ampicillin, gentamicin, or cefotaxime on the day of birth or day 1 or 2 after birth. ^e^Antibiotics for >3 days, proportion of infants with negative blood culture and treated with ampicillin, gentamicin, or cefotaxime for longer than 3 days duration; Infants with early onset sepsis or dead in the first three days of life were excluded (n = 21)*.

### Evolution of Process Indicators Over Time

The percentage of newborns who were not administered antibiotics (**no antibiotics**) between birth and discharge increased, regardless of newborn characteristics, from 6% in 2014 to 30% in 2019 (trend test, *p* < 0.001) (Table 1).

**Figure 1:**
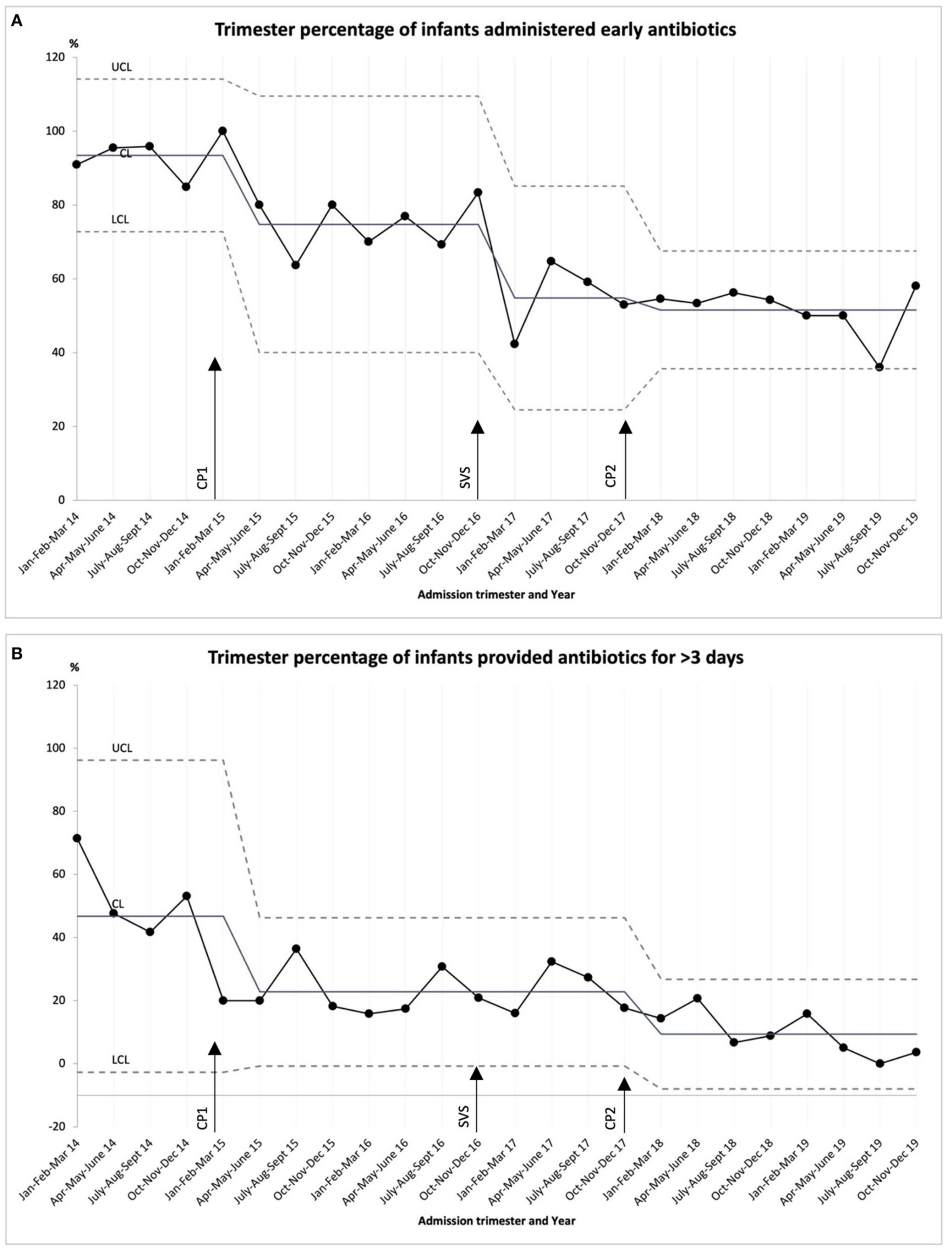
Statistical process control (SPC) chart for VLBW admitted to the NICU between 2014–2019. **(A)** Trimester percentage of infants administered early antibiotics (ampicillin, gentamicin, or cefotaxime on the day of birth or days 1 or 2 after birth). Mean before clinical practice change (CP) 1 (93%) and after CP 2 (52%). (B) Trimester percentage of infants provided antibiotics for longer than 3 days (infants with negative blood culture who were treated with ampicillin, gentamicin, or cefotaxime for longer than 3 days duration). Mean before CP1 (47%) and after CP2 (9%). CP 1: Early suspension, antibiotics (ampicillin, gentamicin, or cefotaxime) started at birth should be suspended at 72 h upon negative blood culture. CP 2: No antibiotics for VLBW with low risk of EOS— Antibiotics should not be started after birth if all the following are present: birth by cesarean delivery, absence of signs of chorioamnionitis, absence of any rupture of membranes before delivery. Surveillance system (SVS): monitoring the use of antibiotics in VLBW through process indicators related to antibiotic use. Dashed lines indicate the upper control limit and lower control limit: +3σ/−3σ (SD), center line: mean.

The proportion of infants provided **early antibiotics** decreased from 91% in 2014 to 49% in 2019 (trend test, *p* < 0.001) (Table 1). SCV was noted on the SPC (Figure 1A) in three occasions and the CL was adjusted accordingly. The first SCV was noted from January 2015 to March 2015 with four of five successive points lying beyond 1 SD from the mean. The change was attributed to the clinical practice change related to early suspension of antibiotics. The second SCV was noted in January of 2017 with one point outside the lower control limit and was attributed to the launch of the surveillance system in the NICU. Finally, a third SCV was noted from January to March of 2018 with four out of five successive points lying at more than 1 SD from the mean on the same side of the CL, this change was attributed to clinical practice change related to no antibiotics for VLBW with low risk of EOS.

There was also a reduction in the number of patients prescribed **antibiotics for longer than 3 days**, from 53% in 2014 to 5% in 2019 (trend test, *p* < 0.001) (Table 1). On the corresponding SPC (Figure 1B), SCV was noted in two occasions and the CL was adjusted accordingly. Similar to the results with early antibiotics, a SCV was noted from January 2015 to March 2015 with four of the five successive points lying beyond 1 SD from the mean and was also attributed to the clinical practice change related to early antibiotic suspension. Another SCV was noted from January to March of 2018 with eight successive points on the same side of the CL, this variation corresponds to clinical practice change related to no antibiotics for VLBW with low risk of EOS.

### Safety: Evolution of Morbidity and Late Mortality, LOS, and Hospital Stay Over Time

Safety analysis monitored morbidity and late mortality over time, and we excluded those who died before 3 days of life and those who were treated for EOS (*n* = 21). Thus, data from the remaining infants (*n* = 567) were used in the analysis (Table 2).

**Table 2:** Evolution of morbidity and mortality, LOS, and hospital stay between 2014–2019.

	**2014**	**2015**	**2016**	**2017**	**2018**	**2019**	**2014–2019**	***p*-value^**a**^**
	***N*** **=** **98**	***N*** **=** **90**	***N*** **=** **91**	***N*** **=** **97**	***N*** **=** **100**	***N*** **=** **91**	***N*** **=** **567**	
*Outcome*								
Morbidity-mortality ^b^	25/98 (26%)	18/90 (20%)	17/91 (19%)	22/97 (23%)	21/100 (21%)	23/91 (25%)	126/567 (22%)	0.9
Late mortality ^c^	13/98 (13%)	5/90 (5%)	6/91 (6%)	6/97 (6%)	4/100 (4%)	3/91 (3%)	37/567(7%)	0.01
BPD	5/89 (5%)	7/86 (8%)	4/85 (4%)	4/91 (4%)	10/96(10%)	9/88 (10%)	39/535(7%)	0.1
ROP	1/86 (1%)	1/86 (1%)	3/86 (3%)	4/94 (4%)	4/96 (4%)	10/91 (10%)	23/539 (4%)	<0.001
Brain Injury	4/88 (5%)	4/87 (5%)	5/89 (6%)	5/92 (5%)	4/99 (4%)	7/91 (7%)	29/546 (5%)	0.4
NEC	12 (12%)	3 (3%)	4 (4%)	5 (5%)	3 (3%)	3 (3%)	30 (5%)	0.01
LOS	31 (30%)	28 (30%)	37 (29%)	23 (23%)	24 (23%)	27 (29%)	170 (30%)	0.2
Length of stay (SD)	66 (35)	64 (34)	70 (41)	72 (31)	62 (27)	66(34)	65 (37)	0.22

*BPD, bronchopulmonary dysplasia: oxygen at 36 weeks' postmenstrual age or at discharge; ROP, retinopathy of prematurity stage ≥ III or those that needed treatment; NEC, necrotizing enterocolitis Bell's Stage ≥ II; LOS, late onset sepsis; SD, standard deviation. ^a^ p-value corresponds to a trend analysis for categorical variables and an analysis of variance for continuous variables for the period 2014–2019. ^b^ Morbidity-mortality: composite outcome of late mortality and 1 of 4 major morbidities (BPD, ROP stage ≥ III or those treated, NEC and brain injury). Infants with early onset sepsis or dead in the first three days of life were excluded from this analysis (n = 21). ^c^ Late mortality: death between 72 h of life and discharge*.

No significant changes in morbidity or late mortality were identified during analysis of the composite variable or each of its components, except for the decrease in the incidence of NEC (p = 0.01) and late mortality (*p* = 0.01) between the first year and the following years. An increase in the incidence of ROP (stage ≥ III or those treated in the last year) (*p* < 0.001) was also noted. No differences over time were seen in LOS or hospital stay.

## Discussion

This follow-up study of a VLBW cohort over a 5-year period demonstrated an improvement in antibiotic prescribing patterns in the context of a QI initiative that is in line with EU Guidelines for the prudent use of antimicrobials (11). Using a low complexity measure to monitor indicators related to antibiotic use, we found that, compared to 2014, there was a significant decrease in the use of early antibiotics and in antibiotics for longer than 3 days by 2019. Additionally, an increase in the number of VLBW not provided any antibiotic therapy was also seen during this period.

As optimal use of antibiotics in newborns is critical, it is essential that all NICUs have an easy and affordable method of measuring the use of antibiotics over time. Based on multicollaborative projects (13, 18) within the QI initiative, we propose a simple and reproducible method to monitor the use of antibiotics as a further step to improve the quality of care for newborns.

Antibiotic use is often measured through exposure rates, which calculate the number of days in which a patient receives one or more antibiotics per 100 patient-days. It can also be measured as duration of exposure wherein the number of antibiotic doses received is multiplied by the interval and divided by 24 h. Although, both methods allow thorough control of the antibiotics administered, monitoring this data can be tedious if an electronic medication prescription and recording system is not in place. Consequently, NICUs that do not have automated data collection can benefit from the use of a simpler monitoring system, such as the one applied in our unit and described here, wherein indicators are measured in percentages and process indicators are defined with yes/no answers.

During the study period, three interventions were introduced, namely, implementation of an antibiotic use surveillance system and improvement of two departmental protocols. Strikingly, these practical changes related to early suspension of antibiotics had a positive effect not only on **antibiotics for longer than 3 days** but on **early antibiotics**. We believe that this effect is due to the incorporation of the concept of prudent use of antibiotics in the NICU. Additionally, implementation of the surveillance system (daily audit, patient report, coding, and annual feedback) in 2017 had a significant effect on **early antibiotics**, which was carried over to the second clinical practical change, namely, no antibiotics for VLBW with low risk of EOS. However, the effects of both interventions overlapped in the beginning of 2018 when the first annual feedback was obtained and shared with the unit's staff. The evolution of the indicators over time reflects the fact that these interventions reinforce each other and help reduce antibiotic use. We believe that the high acceptance rate of the measures implemented in our unit is related to their low cost and simplicity, as well as to the culture of change established in our NICU.

The increase in ROP requiring treatment during 2019 is not attributable to the changes in antibiotic use; rather, it may be due to a change of the indications of ROP treatment with the new anti-vascular endothelial growth factor. Therefore, the number of infants treated increased but not the severity of ROP per se.

Our results concur with those obtained by other units after implementing QI programs to reduce antibiotic use. Compared to the results of the Pediatrix group (80 to 74%, 2007–2013) we report a greater reduction in the use of **early antibiotics** for the period 2014–2019, i.e., from 91 to 50%. Likewise, **antibiotics for longer than 3 days** decreased from 53 to 5% compared to the 35 to 28% reported by the Pediatrix group (18).

One of the NICUs participating in the Vermont Oxford Network's “Choosing Antibiotics Wisely” initiative (25) published its results individually after participating in the program (17). Over a 4-year period, they implemented 10 clinical interventions, including four departmental protocols and dissemination of the antibiotic use rate. They then analyzed process indicators, some of which are identical to those used in our NICU. They obtained a relative reduction of 17% (95 to 70.2%) in **early antibiotics** in infants born at <35 weeks, and doubled the percentage of infants discharged without receiving antibiotics (**no antibiotics**, 15.8 to 35%). In our cohort, these values were, respectively, 47% and a fivefold increase. Additionally, that NICU achieved its goal of reducing antibiotic use rate by 20% and this has remained stable over time. The authors state that the observed variations in antibiotic use rate over time correspond to shifts in process indicators and conclude that both measures facilitated a positive impact on their main objective. They also conclude that their results are due to several factors such as frequent audits with feedback, dissemination of antibiotic use rate, and staff education.

Although our study design did not include antibiotic use rate, additional studies focused on quality indicators can help achieve favorable results in other units' efforts to optimize antibiotic use.

We can learn from previous QI initiatives and stewardship programs that including multiple key elements, NICUs can reduce their antibiotic use, but most importantly is that the shift on the thinking on antibiotics, it is what strengthens those initiatives (2, 13–15, 17). Leadership, multidisciplinary, training and education, development of local guidelines, are actions that contributes to sustain a NICU's culture change.

With so many factors justifying or limiting antibiotic use, a crucial issue is the break-even point that NICUs should achieve (20, 26). It is possible to greatly reduce antibiotic exposure, which could be beneficial for our patients, but “optimal” antibiotic use rate has not yet been identified. Moreover, although there are no recommendations on specific targets, there are guidelines at the European level, with procedures and measures to be considered in programs focused on optimizing antibiotic use (11). Additionally, there are reference values of antibiotic use indicators from multicollaborative neonatal networks (13, 18) that allow any NICU to develop stewardship programs and compare their own results with those available from other units.

In conclusion, with this initiative, we propose a simple and low-cost method to reduce antibiotic use. Through a QI initiative that includes elements described in European guidelines, and by comparing our results with those available for other networks, we have achieved a significant decrease in the use of antibiotics in VLBW infants. To date, there is no registry in Spain that includes the use of antibiotics in NICUs; hence, this aspect has been considered as a quality process indicator by other groups and has been included in the European Guidelines as one of the core elements for optimization of antimicrobial use (11). Its inclusion as a quality indicator on a national basis is expected to encourage the establishment of restrictive antibiotic prescribing policies, as well as the creation of a national multicollaborative network to evaluate procedural differences between units.

## Data Availability Statement

The original contributions presented in the study are included in the article/supplementary material, further inquiries can be directed to the corresponding author/s.

## Ethics Statement

The studies involving human participants were reviewed and approved by Ethics Committee of the 12 de Octubre University Hospital. Written informed consent from the participants' legal guardian/next of kin was not required to participate in this study in accordance with the national legislation and the institutional requirements.

## Author Contributions

CM-B, CP-A, and JD conceptualized the design of the study, coordinated and supervised data collection, drafted the initial manuscript, and reviewed the manuscript. BM-A and EB-S contributed to the acquisition, analysis, interpretation of data, and reviewed the manuscript. All authors contributed to the article and approved the submitted version.

## Conflict of Interest

The authors declare that the research was conducted in the absence of any commercial or financial relationships that could be construed as a potential conflict of interest.

## Publisher's Note

All claims expressed in this article are solely those of the authors and do not necessarily represent those of their affiliated organizations, or those of the publisher, the editors and the reviewers. Any product that may be evaluated in this article, or claim that may be made by its manufacturer, is not guaranteed or endorsed by the publisher.
